# Histological, cellular and behavioural analyses of effects of chemotherapeutic agent cyclophosphamide in the developing cerebellum

**DOI:** 10.1111/cpr.12608

**Published:** 2019-04-01

**Authors:** Yu Zhang, Yongfang Li, Wenqin Luo, Yaohui Tang, Jia Wang, Ru Yang, Wei‐Qiang Gao

**Affiliations:** ^1^ State Key Laboratory of Oncogenes and Related Genes, Renji‐Med X Clinical Stem Cell Research Center, Ren Ji Hospital, School of Medicine Shanghai Jiao Tong University Shanghai China; ^2^ School of Biomedical Engineering & Med‐X Research Institute Shanghai Jiao Tong University Shanghai China; ^3^ Department of Neurology, Ruijin Hospital, School of Medicine Shanghai Jiao Tong University Shanghai China

**Keywords:** cerebellar development, cyclophosphamide, granule neuron progenitors, Math1, replenishment

## Abstract

**Objectives:**

We performed histological, cellular and behavioural analyses of the effects of cyclophosphamide (CTX), a chemotherapeutic drug, in the developing cerebellum and aimed to provide valuable insights into clinical application of CTX in children.

**Materials and methods:**

C57BL/6 mice and Math1‐dependent GFP expression transgenic mice were used in the research. H&E staining was performed to analyse histological effects of CTX in the cerebellum. Staining for EdU and TUNEL was used to estimate the cell proliferation and apoptosis. Rotarod test and hanging wire test were used to evaluate the behavioural functions. Immunofluorescent staining was used to identify the cell types. The differentiation markers and genes related to Sonic Hedgehog (SHH) signalling were measured via quantitative real‐time PCR or immunoblotting.

**Results:**

We found that while CTX induced a significant reduction in cell proliferation and increased apoptosis in the EGL in 48 hours, the behavioural functions and the multilayer laminar structure of cerebella were largely restored when the mice grew to adults. Mechanistically, granule neuron progenitors, driven by the SHH signalling, enhanced the capability of proliferation quickly after CTX administration was stopped, which allowed the developing cerebellum to catch up and to gradually replenish the injury.

**Conclusion:**

The chemotherapeutic agent CTX induces an immediate damage to the developing cerebellum, but the cerebellar multilayer laminar structure and motor function can be largely restored if the agent is stopped shortly after use.

## 1 INTRODUCTION

Cancers are serious diseases threatening human health and life. Chemotherapy is often one of the options for the management of various types of cancers. Not only adults, but also many children suffer from cancers. Nowadays, more and more children can survive from cancer by chemotherapy.^1^ However, cytotoxic side effects of chemotherapeutic agents on healthy tissues have been a major concern, in particular, how severe they affect developing cerebella in children is often unclear.

Cyclophosphamide (CTX), an alkylating agent, among the most widely used chemotherapeutic agents has a good application in a variety of malignant and non‐malignant tumours in both children and adult patients.^2^ In the liver, CTX is metabolized into a number of compounds. Phosphoramide mustard, one of these compounds, binds to DNA and prevents it from replicating and then stops cancer cells from proliferating.^3^ In addition to cancers, it is also applied to treat serious systemic autoimmune diseases such as rheumatoid arthritis, systemic lupus erythematosus and multiple sclerosis,^4^ as well as in prevention from graft rejection following transplantation surgery.^5^ In spite of its wide usage, CTX causes a wide range of adverse effects including immunosuppression,^6^ bone marrow suppression, gonadal toxicity, leucopaenia and oxidative stress.^4^, ^7^, ^8^

Different from other regions of the brain, the cerebellum undergoes its major developing period from the third trimester to infant stage in humans.^9^ As a result, the cerebellum is highly prone to injury in children.^10^ The developing cerebellar cortex is a simple layered structure.^11^ After birth, granule neuron progenitors (GNPs) expand rapidly in the cerebellar external granule layer (EGL). GNPs exit the cell cycle and migrate towards the internal granule layer (IGL) through the underlying molecular layer (ML) and the Purkinje cell layer (PCL). Then, they complete their differentiation programme.^12^, ^13^, ^14^ The period of granule cell neurogenesis coincides with critical sensitivity to the environment.^15^, ^16^ Due to the unique characteristics of proliferation of GNPs postnatally, CTX may have strong toxicity on the developing cerebellum in children. Up to now, however, whether and how CTX affects progenitor cell proliferation and differentiation during the cerebellar development have not been determined.

Here, we set forth to determine possible effects of CTX on the mouse developing cerebellum. We used histological, cellular and behavioural methods to examine possible changes in the cerebellum after the challenge by CTX. While CTX induced a significant reduction in cell proliferation and increased apoptosis in the EGL in 48 hours, the behavioural functions including motor coordination and balance and the multilayer laminar structure of cerebella were largely restored when the treated mice grew to adults. Mechanistically, GNPs, driven by the Sonic Hedgehog (SHH) signalling, enhanced the capability of proliferation quickly after CTX treatment was stopped, which allowed the developing cerebellum to catch up and to gradually replenish the injury. This study would provide valuable insights into clinical application of CTX in children.

## 2 METHODS

### 2.1 Animal and treatments

In addition to C57BL/6 mice, a transgenic mouse line Math1‐GFP (in which GFP expression is driven by the Math1 promoter) was used in the present research.^17^, ^18^ Mice used in all these experiments were raised in the specific pathogen‐free animal centre at Renji Hospital, Shanghai Jiaotong University. The temperature and humidity were controlled. All animal experimental protocols were approved by the Animal Research Ethics Committee of Renji Hospital. A single intraperitoneal injection of CTX (50 mg/kg) was administrated to mice at postnatal day 6 (P6). Mice were sacrificed at indicated ages.

### 2.2 Histology and immunofluorescence

Cerebella were collected and submersion fixed in 4% paraformaldehyde overnight at 4°C and then dehydrated in 30% sucrose. Tissues were embedded in optimal cutting temperature (OCT) compound (Sakura Finetek) before frozen and sectioned in sagittal plan on a Leica cryostat at 6 μm. Sections were stained with haematoxylin and eosin. For immunofluorescent staining, the slides were first treated with 0.5% Triton X‐100 for 10 minutes and blocked with 10% donkey serum for 1 hour at room temperature. After blocking, slides were incubated overnight at 4℃ with the following primary antibodies which were diluted in PBS containing 1% goat serum: rabbit anti‐Sox2 (Abcam, ab92494), mouse anti‐Calbindin‐D28K (Sigma Aldrich, C9848) and rabbit anti‐Olig2 (Abcam, ab109186). Primary antibodies were rinsed in PBS for three times with 5 minutes each time. After extensive washing, secondary antibodies, conjugated to Alexa‐488, 594 or Cyanine‐Cy3 (Life Technologies), were applied and the slides were incubated for 1 hour at room temperature. Secondary antibody was rinsed with PBS three times before the slides were mounted. After finishing washing secondary antibody, slides were mounted with mounting medium containing DAPI (Sigma).

### 2.3 RNA isolation and quantitative RT‐PCR

We used TRIzol reagent (Invitrogen) to extract total RNA following the manufacturer's instruction. The cDNA syntheses were reverse‐transcribed from total RNA with PrimeScript RT reagent (Takara, Dalian, China). Quantitative PCR was performed using SYBR Green Real‐time PCR Supermix (Toyobo, Osaka, Japan) and on the Step one Plus RT‐PCR Systems (Applied Biosystems, Waltham, MA, USA). The primers used are accessible in Table S1.

### 2.4 Immunoblotting

Cerebella samples were lysed using RIPA (Pierce, Waltham, MA, USA) containing the protease inhibitor cocktail (Thermo Scientific, Waltham, MA, USA). Proteins were separated in SDS‐PAGE gels and then transferred to nitrocellulose membranes. Membranes were incubated with primary antibody overnight at 4°C after blocked with 5% fat‐free milk in TBS for 1 hour at room temperature. After washed three times in TBS containing 1% Tween 20, the membranes were then incubated with horseradish peroxidase‐conjugated secondary antibody for additional 1 hour. Antibodies against Gli1, Gli2, N‐Myc and β‐actin were purchased from Cell Signaling Technology (2643), Aviva Systems Biology (ARP31885_P050), Santa Cruz (sc‐53993) and Cell Signaling Technology (4970), respectively.

### 2.5 EdU assays

Mice at designated ages received intraperitoneal injections of EdU (5 mg/kg) 1 hour before sacrifice for analysis of proliferation. After cryostat sectioning, EdU was detected following the manufacturer's protocol.

### 2.6 TUNEL staining

The experiment was performed using a nick end labelling kit (Roche, Penzberg, Germany), and the procedure was according to the introductions. We incubated the sections with 50 μL TUNEL mixture (5 μL enzyme solution and 45 μL labelling solution) for 3 hours at room temperature. The sections were stained with 0.1 g/mL DAPI (Sigma) for five minutes before mounted.

### 2.7 Rotarod test

We assessed the effect of CTX treatment on motor coordination and balance using a rotarod test, which was performed with a rotarod apparatus. The ability of a mouse to balance was evaluated on a rotating rod. As the speed of rotation was increased, it would be more difficult for the mouse to keep its balance. We set rotating speed and cut‐off time at 40 rpm and 3 minutes, respectively. The latency to fall off the rotating rod was recorded within the time period and used as an indication of motor coordination and balance. Briefly, the mice were acclimatized for 4 days and on the fifth day tests were carried out. We gave the mice three trials at the predefined speed level and measured the time to drop off the rod taken by each mouse. The incidence of ataxia, that is the ability of the mouse to fall, was recorded in different experimental groups. The differences between groups were statistically analysed by Student's t test.

### 2.8 Hanging wire test

We performed another test called hanging wire test to achieve a measurement of motor function using a rating scale and evaluate the deficit of CTX treatment mice. The test began with the animal hanging from a 55 cm wide metal wire. Initially, each mouse had a score of 10. The animal was placed on the middle of the elevated wire and suspended on the wire. In this method, mice were subjected to a 180 seconds lasting hanging test. When a mouse fell or reached one of the sides of the wire, the score was diminished or increased by 1, respectively. We created a Kaplan–Meier‐like curve afterwards. This test was performed three days per week with three trials per session. The average performance for each session was presented as the average of the three trials. The differences between groups were statistically evaluated by Student's *t* test.

### 2.9 Statistical analysis

All the experimental data were analysed and expressed as mean ± SD. Student's *t* test was used for statistical analysis. *P *values <0.05 were considered to have statistical significance. All statistical analyses were performed using GraphPad Prism statistical version 7.

## 3 RESULTS

### 3.1 Postnatal intraperitoneal injection of CTX results in an immediate, major loss of the EGL

**Figure 1:**
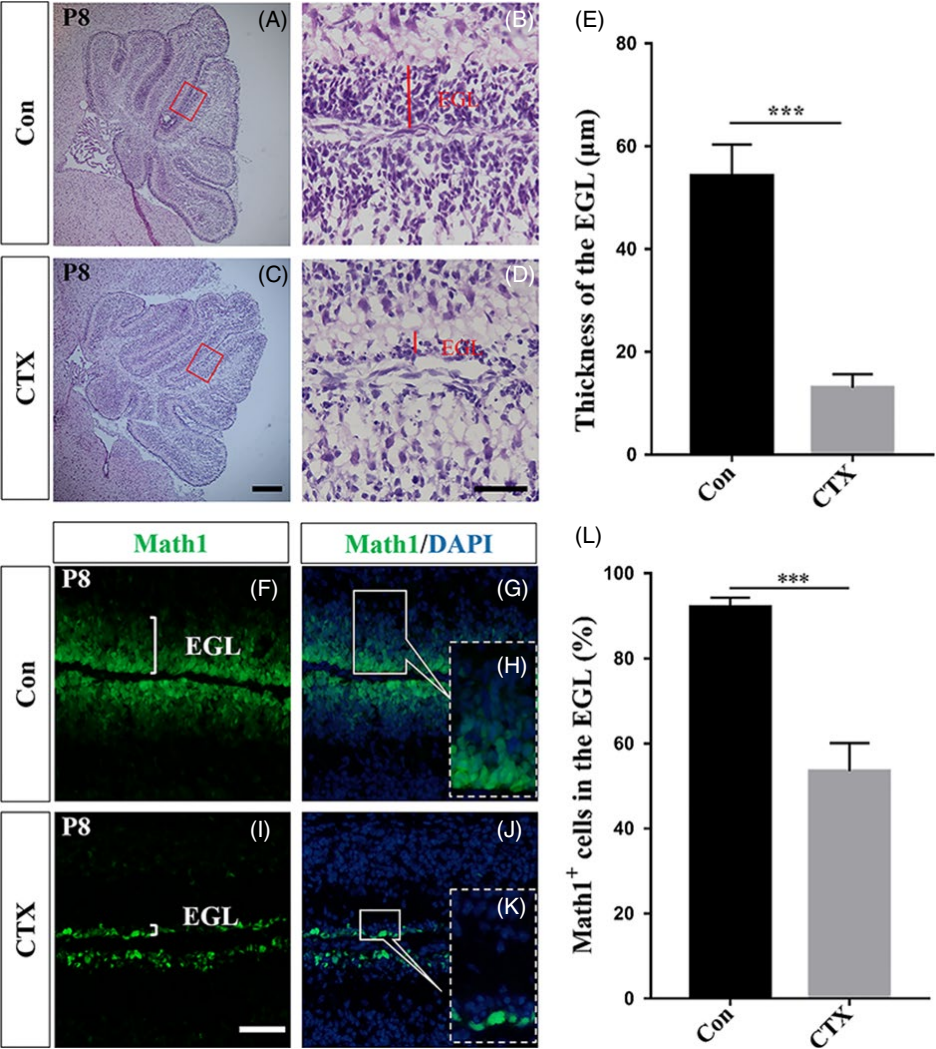
Postnatal intraperitoneal injection of CTX results in an immediate, major loss of the EGL. (A‐D) Haematoxylin and eosin (H&E) staining on midsagittal sections of CTX‐treated (C, D) and PBS‐treated mice (A, B) at p8, 48 h post‐injection. (A, C) CTX‐treated cerebella lose almost complete EGL (red rectangles. Scale bar, 200 μm). (B, D) High‐power images of the areas indicated by red rectangles in A and C (Scale bar, 50 μm). (E) Graph of the thickness of EGL of CTX‐treated and PBS‐treated cerebella at P8, n = 3, *P* < 0.001. (F‐K) Fluorescence immunohistochemistry detection of the Math1 and DAPI on sections of PBS‐treated and CTX‐treated mice at P8. Scale bar, 50 μm. (H, K) Representative and high‐power images from G and J. The smaller number of Math1^+^ cells strongly suggests that nearly all EGL cells are depleted following CTX treatment. (L) Graph of the proportion of Math1^+^ cells in both groups, n = 3, *P* < 0.001

To determine possible neurotoxic effects of CTX on newborn mouse cerebella, we first assessed possible histological changes in the cerebellar EGL at the stage of cerebellar development following administration of CTX. While with high concentration (100 mg/kg), the mice could not survive to adulthood, we specifically gave a single intraperitoneal injection (50 mg/kg) of CTX or PBS^9^ as a control to mice at postnatal day 6 (P6). Both PBS‐treated (Con) and CTX‐treated (CTX) mice were sacrificed at P8, 48 hours after the injection. The EGL was examined by haematoxylin and eosin staining (H&E staining) (Figure 1A‐D) as well as for GNP marker Math1^+^ cells (Figure 1F‐K).^17^, ^18^, ^19^, ^20^, ^21^ Math1‐GFP transgenic mouse line was used to detect Math1 expression rather than using an antibody against Math1.^17^, ^18^ Math1^+ ^layer was regarded as the EGL.^17^, ^18^ H&E and Math1 staining at P8 revealed a high sensitivity of the EGL to CTX (Figure 1C, D, I, J and K) compared to the EGL in PBS‐treated mice (Figure 1A, B, F, G and H). The EGL was greatly diminished at P8 (Figure 1E, n = 3, *P* < 0.001). Consistently, analysis of the Math1‐GFP mouse cerebella also revealed a significant decrease in the number of Math1^+^ cells in the EGL (Figure 1L). In short, postnatal intraperitoneal injection of cyclophosphamide at P6 mice resulted in an immediate, major loss of the EGL by P8 based on histological and immunofluorescent staining.

### 3.2 CTX reduces the number of proliferating cells significantly and increased cell death in the EGL

**Figure 2:**
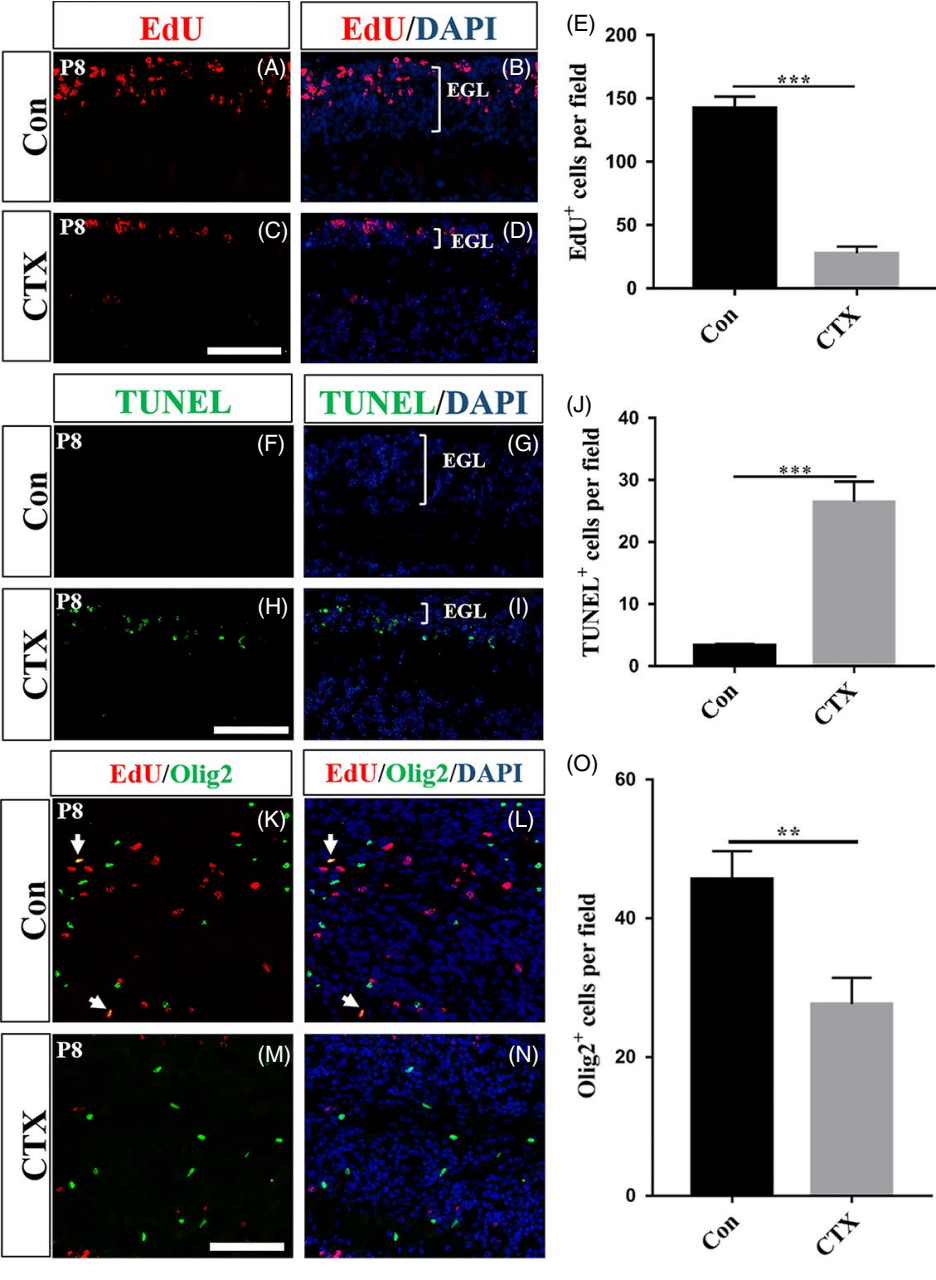
CTX reduces the number of proliferating cells significantly and increased cell death in the EGL. (A‐D) EdU staining in the EGL of cerebellar sections at P8, 48 h after PBS‐treated or CTX‐treated. (C, D) CTX sharply reduces proliferating cells, as revealed by the presence of a smaller number of EdU^+ ^cells. (E) Graph of the number of EdU^+^ cells per high field in PBS‐treated and CTX‐treated sections (n = 3, *P* < 0.001). (F‐I) Detection of the TUNEL and DAPI on sections of PBS‐treated and CTX‐treated mice at P8. (L) Graph of the number of TUNEL^+^ cells per field in each group sections. (n = 3, *P* < 0.001). (K‐N) EdU and Olig2 immunostaining in the IGL of both groups at P8. The cells double stained of EdU and Olig2 are indicated in white arrows. (O) Graph of the number of Olig2^+^ cells per field in both group sections (n = 3, *P* < 0.01). Scale bar, 50 μm

To find out cellular basis for the histological changes in the cerebellum induced by CTX, we examined cell proliferation and apoptosis. We gave the mice EdU by intraperitoneal injection 1 hour before the animals were sacrificed to determine a possible difference in the number of proliferating cells between PBS‐ and CTX‐treated mice at P8. Proliferating cells were labelled by EdU staining. As shown in Figure 2E, EdU^+^ cells were significantly decreased in CTX‐treated sections (Figure 2C and D, n = 3, *P* < 0.001), indicating that CTX had a strong toxic effect on the proliferation of cells in the EGL during cerebellar development. Meanwhile, much more apoptotic cells were found in the EGL of the CTX‐treated mice based on in situ TUNEL staining (Figure 2H and I), compared to that in the PBS‐treated mice (Figure 2F and G, Figure 2J, n = 3, *P* < 0.001). These results provided a cellular mechanism for the thinner EGL in the CTX‐treated animals described above. To find out whether CTX also affects proliferation and cell death of other cerebellar cells in addition to the cells in the EGL, we performed immunostaining for oligodendrocyte marker Olig2 and Purkinje cell marker Calbindin. As shown in Figure 2K‐O, CTX also reduced the number of proliferating oligodendrocytes in the IGL at P8 without changes in their apoptosis (Figure S2a‐d). For Purkinje cells in the PCL, we did not find any apparent impact caused by CTX (Figure S1a‐n) at P8 or at P10 except for occasional dislocation in the CTX‐treated sections (Figure S1k and l). Considered together, chemotherapeutic drug CTX can induce a significant reduction in the number of proliferating cells and lead to increased apoptosis in the EGL without an apparent toxicity on Purkinje cells during cerebellar development.

### 3.3 Motor coordination and balance behaviour tests do not show significant defects in the mice 8 weeks after CTX challenge

**Figure 3:**
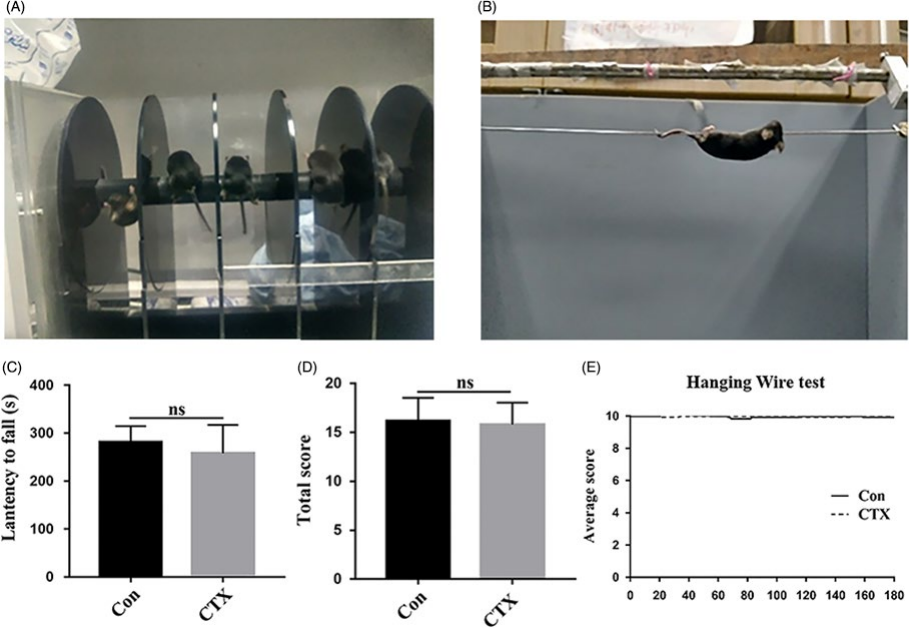
Motor coordination and balance behaviour tests do not show significant defects in the mice 8 wk after CTX challenge. (A) Device used in the rotarod test. (B) The picture of performing hanging wire test. (C) Graphs representing the latency to fall for each trial or total of PBS‐treated (n = 12) and CTX‐treated (n = 16) mice, *P* = 0.2. (D) Graphs representing the hanging wire test—total score of control (n = 12) and CTX‐treated (n = 16) mice, *P* = 0.9. (E) A Kaplan–Meier‐like curve created according to the results of the hanging wire test. There is no statistic difference in the two groups for the motor function test

To investigate whether the significant injury in developing cerebella induced by CTX is temporary or permanent, we performed behaviour tests in the mice 8 weeks following treatment of CTX. We measured motor coordination and balance to assess the effect of CTX on mice using the rotarod test and the hanging wire test (Figure 3A and B). The original data are shown in Tables S2‐S4. No significant difference was observed regarding the accelerating rotarod performance for CTX‐treated mice (n = 16, 258 ± 59.45 s) compared to control mice (n = 12, 281.58 ± 33.17 s, Figure 3C, *P* = 0.2). Similarly, there was a relatively normal grip strength in CTX‐treated mice (n = 16, mean score = 15.81 ± 2.22) compared to control ones (n = 12, mean score = 15.90 ± 1.30) mice (Figure 3D and E, *P* = 0.9). These data showed that CTX does not significantly disrupt motor coordination and balance in grow‐ups although CTX treatment caused a major loss of EGL and a decrease in proliferating cells at an early stage.

### 3.4 CTX reduces cerebellar sizes significantly but the multilayer laminar structure of cerebella is resumed

**Figure 4:**
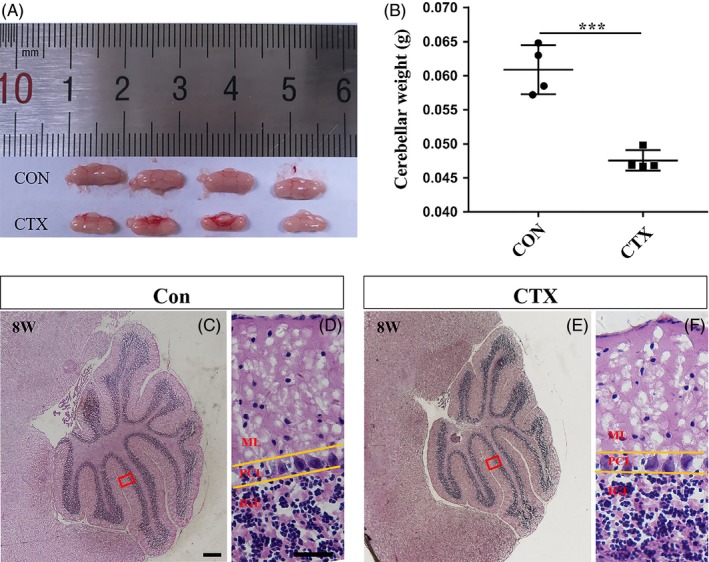
CTX reduces cerebellar sizes significantly but the multilayer laminar structure of cerebella is resumed. (A) The picture of the cerebella from PBS‐treated (n = 4) and CTX‐treated (n = 4) mice at 8 wk. (B) Graph of the weight of cerebella of both groups at 8 wk, CTX‐treated cerebella are smaller and lighter in weight, *P* < 0.001. (C, E) Haematoxylin and eosin (H&E) staining on midsagittal sections of each group. Scale bar, 200 μm. (D, F) High‐power images of the areas indicated by red rectangles in c and e, scale bar, 20 μm

Given the facts that CTX induced a histological deficit of EGL and cellular proliferation and that long‐term behaviour tests with rotarod and hanging wire did not show a dysfunction in motor coordination and balance, we decided to examine cerebellar size and laminar layer structure. After completion of the motor function assays, the cerebella were dissected out and processed for histology from CTX‐treated and PBS‐treated mice and weighed. There was an apparent difference between the two groups, that is the size of the cerebellum from the CTX‐treated group was significantly smaller (Figure 4A and B). However, histological analysis revealed that the multilayer laminar structure was well‐formed (Figure 4E and F), including the ML, the PCL and the IGL, implicating that the neural circuit appears largely restored and the function is not persistently affected. Taken together, once the drug treatment is stopped, there appears a catch‐up repair to compensate the early defect in developing cerebella.

### 3.5 The restoration of the CTX‐induced deficit in the EGL is achieved mainly by an increased number of proliferating Math1^+^ GNPs

**Figure 5:**
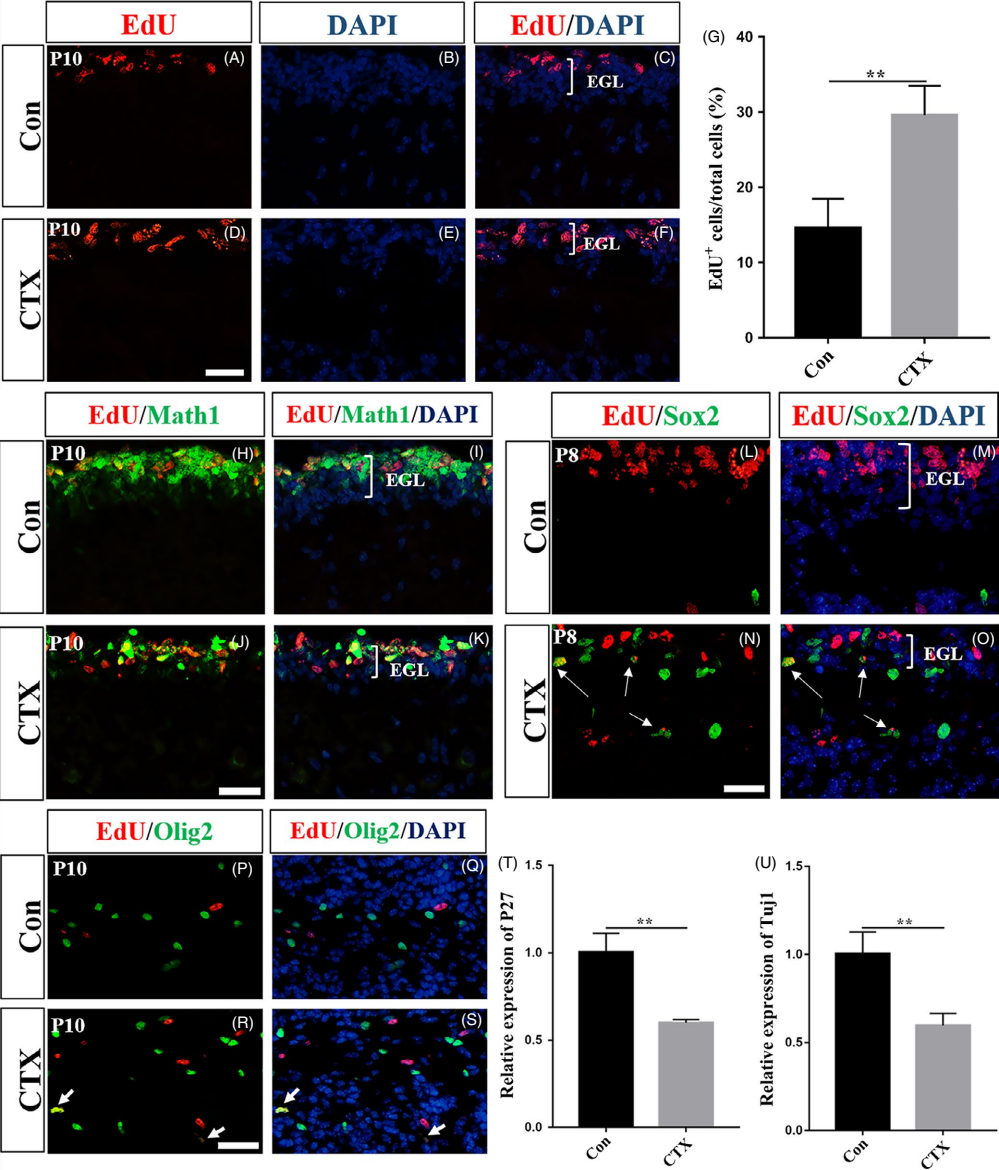
The restoration of the CTX‐induced deficit is achieved by an increased number of proliferating Math1^+^ GNPs, Sox2^+^ cells and Olig2^+^ oligodendroglial lineage cells. (A‐F) Detection of the EdU and DAPI on midsagittal cerebellar sections in both groups at P10. (G) Graph of the percentage of EdU^+^ cells in PBS‐ and CTX‐treated cerebella at P10, n = 3, *P* < 0.01. (H‐K) Math1 and EdU staining. Most of the proliferating cells in the EGL were Math1‐positive cells in both groups at P10. (L, M) Only few Sox2^+^ cells were found in the PBS‐treated section, and they appeared in the PCL and IGL. (N, O) A lot more Sox2^+^ cells were found in all layers in the CTX‐treated sections, and there were proliferating ones in the EGL (white arrows). (P‐S) Immunostaining for Olig2 and EdU at P10. (R, S) More proliferating Olig2^+^ cells were found in the IGL in the CTX‐treated group than PBS‐treated group. (T, U) Quantitative real‐time PCR analysis showed a significantly lower expression of P27 (*P* < 0.01) and Tuj1 (*P* < 0.01), n = 4, scale bar, 50 µm

A recent report by Daniel N Stephen and his colleagues^12^ indicates that progenitor cells can be adaptively reprogrammed to supplement the post‐injury of cerebellar granule cells after irradiation, which inspired us to suspect that there might be a compensation/catch‐up mechanism for the deficit restoration in the cerebellum. We focused our attention on a possible enhanced ability of GNPs to proliferate after CTX treatment is stopped. By analysing the proportion of EdU labelling cells, the CTX‐treated cerebella were observed to have more proliferating cells in midsagittal cerebellar sections at P10 (Figure 5D and F), compared to the CTX‐treated mice at P8 (Figure 2C and D). Notably, at the same time points, the proportion of EdU^+ ^cells was increased in the EGL of CTX‐treated mice at P10 (Figure 5G), compared to PBS‐treated controls (Figure 5A and C), suggesting a replenishment of the newly forming EGL. Furthermore, as shown in Figure 5H‐K, the same as in the PBS‐treated mice, in the CTX‐treated cerebella, most proliferating cells were Math1‐positive cells, which represent for GNPs.^18^, ^21^ Moreover, at P8, immunostaining for Sox2, another neuronal stem/precursor cell marker,^22^ also revealed an increased number of Sox2‐positive cells in the EGL and ML of the CTX‐treated mice compared to the control mice (Figure 5L‐O). Interestingly, in the CTX‐treated cerebella, parts of Sox2^+^ cells were proliferating ones (Figure 5N and O), which may participate in the restoration of the deficit in the EGL. In addition, we also found more EdU^+ ^oligodendrocytes in the IGL of the CTX‐treated mice than that in the PBS‐treated mice (Figure 5P‐S). Furthermore, by RT‐PCR technology, we found that the CTX‐treated cerebella expressed less level of the P27 and Tuj1 (Figure 5T and U) than the PBS‐treated ones. Taken together, our results suggested a compensatory ability of the developing cerebella to expand GNPs and Sox2^+^ cells for the replenishment of the EGL, which may take a longer time than 4 days to restore the neuronal deficit.

### 3.6 Increased proliferation appears to be mediated by enhanced SHH signalling

**Figure 6:**
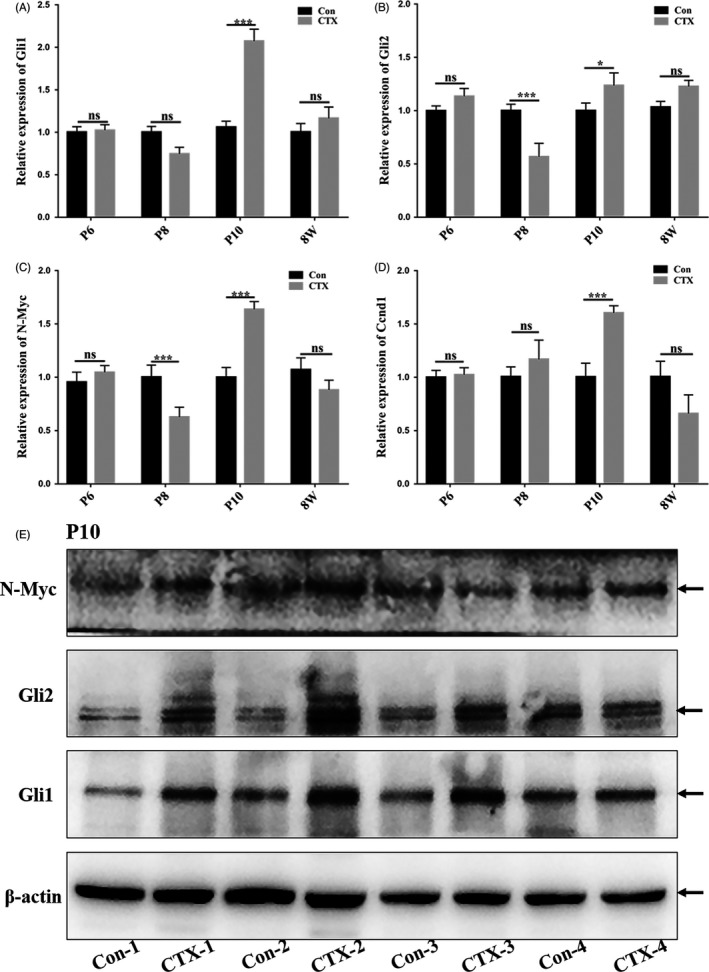
Increased proliferation of cells appears to be mediated by enhanced SHH signalling. (A‐D) RT‐PCR analysis of SHH effector genes and target genes. (A, B) Gli1 and Gli2 were upregulated expressed in the mRNA level at P10 in the CTX‐treated group. (C, D) The expression of SHH target genes N‐Myc and Ccnd1 was upregulated significantly at P10, *P* < 0.001. (E) Immunoblotting of proteins related to SHH signalling at P10 showed that SHH signalling is enhanced to mediate the restoration of the CTX‐induced deficit in the developing cerebella

As an effort to explore the molecular mechanism for the replenishment of EGL, we next performed RT‐PCR to measure possible expression level changes in SHH signalling genes and downstream components, which have been previously shown to control GNP proliferation and modulate the development of cerebellum.^11^, ^23^ As shown in Figure 6A and B, expression of SHH effector genes including Gli1 and Gli2 was significantly upregulated in the CTX‐treated cerebella as compared to the PBS‐treated ones at P10, a stage when we found the restoration was happening. Similarly, expression of SHH signalling downstream components such as Ccnd1 and N‐Myc was also dramatically elevated at P10 (Figure 6C and D). Measurement of the expression level of the proteins related to SHH signalling at P10 indicated that CTX‐treated cerebella had higher expression level of proteins related to SHH signalling (Figure 6E). Therefore, the restoration is likely attributable to upregulated SHH signalling.

## 4 DISCUSSION

In the current study, we for the first time demonstrated that exposure to CTX results in a major loss of cerebellar EGL and a significant decrease in GNP proliferation via immunofluorescent staining. Importantly, a single‐dose neonatal injection of CTX at P6 does not lead to a significant effect on motor coordination and balance in adults based on rotarod test and hanging wire test and cerebellar multilayer laminar structure when the animal become adults via histological examination, in spite of a decrease in cerebellar size. Notably, we found that 96 hours after treatment with CTX, Math1^+^ GNPs accelerates proliferation, which replenishes the EGL deficit and eventually restores the functions of motor coordination and balance in adults.

Although CTX is widely applied in various types of diseases,^24^, ^25^ cytotoxic side effects of CTX on those healthy tissues that are proliferative have been a major concern.^24^, ^25^, ^26^ For example, it has been reported that CTX is toxic to the reproductive system 1 and the haematopoietic system,^26^ which have active proliferation activity. Similarly, there are clinical cases in which CTX is also toxic to the nervous system in both child and adult.^27^, ^28^ Our present study provides detailed analyses for the toxicity of CTX on developing cerebella for the first time.

One unique method used in the current report, which is different from other previous studies, is the behaviour tests, which are directly related to function and quality of life. Although CTX causes a temporal injury, including a significant loss of EGL, a decrease in GNP proliferation and a decrease in cerebellar sizes, our study shows that CTX does not lead to a permanent behaviour deficit, such as motor coordination and balance functions, after a period of recovery from CTX treatment. This result may provide helpful guidance for clinical application of CTX.

It is noteworthy to point out that we found a compensatory mechanism underlying the unharmed motor coordination after CTX treatment.Over the years, little information has been available for the effects of CTX on developing cerebella. The cerebellum is an important part of the central nervous system, which consists of 80% 11 of the neurons in the human brain (60% in mice).^9^, ^12^ It is well known that the cerebellum regulates motor coordination,^24^ and some studies have reported that the cerebellum is also involved in cognitive functions such as language, attention, emotional behaviour and sleep.^29^ In the cerebellum, the most abundant cell type is granule neurons that are differentiated from GNPs.^30^ After birth, GNPs proliferate actively in the EGL.^31^ During this process, the GNPs are sensitive to the damage by toxic substances.^32^ In our study, we found that indeed there is an immediate damage to GNPs in the developing cerebellum following CTX treatment and that cerebellar size was smaller when these mice grew to adults due to the recovery deficit. However, we demonstrated that soon after CTX treatment is stopped, there is a significant increase in the number of proliferating Math1^+^ GNP cells and Sox2^+^ cells. Therefore, the developing cerebella have a compensatory cellular mechanism to attempt to restore the deficit in EGL. Support for our study comes from a previous report indicating that progenitor cells can be adaptively reprogrammed to supplement the post‐injury of cerebellar granule cells after irradiation.^12^ In addition, our RT‐PCR and immunoblotting analyses for SHH signalling molecules indicated that such increased proliferation is attributable to enhanced SHH signalling including upregulation of Gli1 and Gli2, and their downstream components such as N‐Myc and Ccnd1. The results provided the molecular basis for the compensation in the developing cerebella after CTX injury.

In summary, the chemotherapeutic agent CTX has a cytotoxic effect on proliferation of cerebellar GNPs and induces apoptosis as expected. However, if we stop the clinical application of CTX in time, the toxic effect appears limited and the tissues may have a compensatory repair mechanism, so there will only be minimal behavioural defects in adults. A limited time window of CTX treatment should be practiced with cautions to allow a better clinical outcome in children.

## CONFLICT OF INTEREST

There is no conflict of interest in this manuscript.
