# Supplementary figures and images for: Histological, cellular and behavioural analyses of effects of chemotherapeutic agent cyclophosphamide in the developing cerebellum
Source: Cell Prolif. 2019 Apr 1;52(3):e12608. doi: 10.1111/cpr.12608 (PMC6536418; doi:10.1111/cpr.12608)

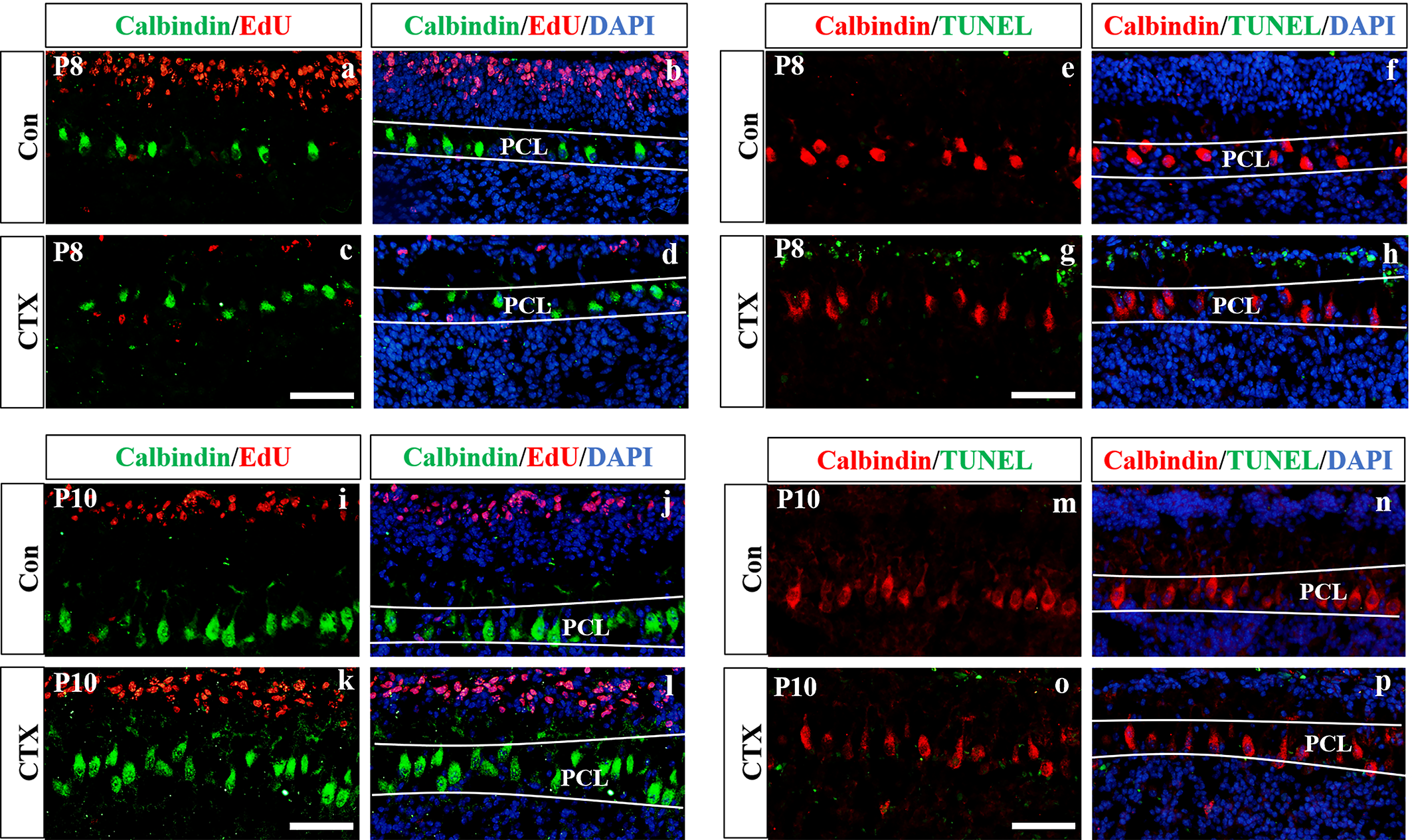

Supplement: Supplementary file 1 [file CPR-52-e12608-s001.tif]

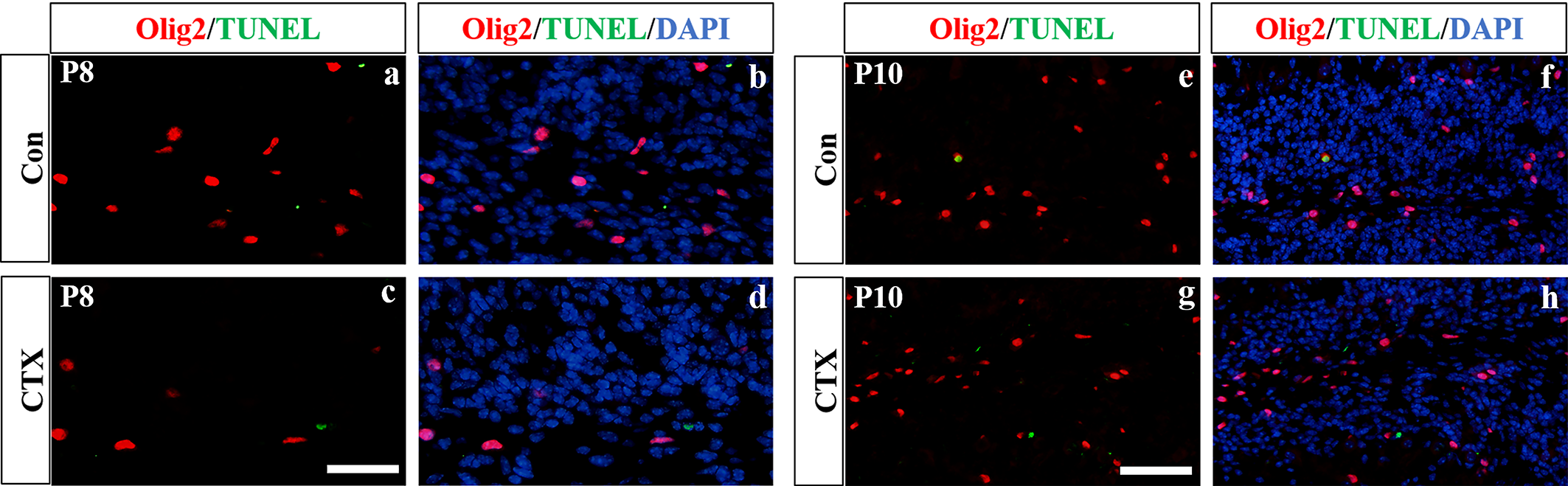

Supplement: Supplementary file 2 [file CPR-52-e12608-s002.tif]
