# Supplementary material for: Histological, cellular and behavioural analyses of effects of chemotherapeutic agent cyclophosphamide in the developing cerebellum
Source: Cell Prolif. 2019 Apr 1;52(3):e12608. doi: 10.1111/cpr.12608 (PMC6536418; doi:10.1111/cpr.12608)
Supplement: Supplementary file 3 [file CPR-52-e12608-s003.docx]

Supplementary table 1. The primers used in RT-PCR assays.

| **Gene** | **Primer Sequence (5' to 3')** |
| --- | --- |
| Tuj1 | F：TAGACCCCAGCGGCAACTAT |
|  | R：GTTCCAGGTTCCAAGTCCACC |
| p27 | F：TCAAACGTGAGAGTGTCTAACG |
|  | R：CCGGGCCGAAGAGATTTCTG |
| Math1-GFP | F：AGGAATTCGATGGGCTGGGCA |
|  | R：CGTCGCCGTCCAGCTCGACCA |
| Gli1 | F：GCTTGGATGAAGGACCTTGTG |
|  | R：GCTGATCCAGCCTAAGGTTCTC |
| Gli2 | F：GCAGACTGCACCAAGGAG |
|  | R：CGTGGATGTGTTCATTGTTGA |
| Ccnd1 | F：GAGCCCAACCGAGACCAC |
|  | R：CTCTTCGCACTTCTGCTCCT |
| N-Myc | F：ATCACTGTACGCCCCAAGAC |
|  | R：CCTTTGGTGGAACGACACTT |
| β-actin | F：GGCTGTATTCCCCTCCATCG |
|  | R：CCAGTTGGTAACAATGCCATGT |

Supplementary table 2. The original data of Rotarod test.

| **Rotarod test** | | |
| --- | --- | --- |
| Group | Animal | Lantency time(sec) |
| Con (n=12) | 1# | 268 |
|  | 2# | 300 |
|  | 3# | 289 |
|  | 4# | 300 |
|  | 5# | 187 |
|  | 6# | 300 |
|  | 7# | 300 |
|  | 8# | 300 |
|  | 9# | 276 |
|  | 10# | 259 |
|  | 11# | 300 |
|  | 12# | 300 |
|  |  |  |
| CTX (n=16) | 1# | 300 |
|  | 2# | 297 |
|  | 3# | 300 |
|  | 4# | 246 |
|  | 5# | 300 |
|  | 6# | 138 |
|  | 7# | 155 |
|  | 8# | 150 |
|  | 9# | 300 |
|  | 10# | 248 |
|  | 11# | 229 |
|  | 12# | 300 |
|  | 13# | 300 |
|  | 14# | 273 |
|  | 15# | 300 |
|  | 16# | 292 |

Con: PBS-treated. CTX: CTX-treated.

Supplementary table 3. The original total score data of Hanging Wire test.

| **Hanging Wire test** | | |
| --- | --- | --- |
| Group | Animal | Total score |
| Con (n=12) | 1# | 13 |
|  | 2# | 16 |
|  | 3# | 18 |
|  | 4# | 16 |
|  | 5# | 17 |
|  | 6# | 14 |
|  | 7# | 12 |
|  | 8# | 15 |
|  | 9# | 15 |
|  | 10# | 18 |
|  | 11# | 19 |
|  | 12# | 18 |
|  |  |  |
| CTX (n=16) | 1# | 13 |
|  | 2# | 14 |
|  | 3# | 18 |
|  | 4# | 15 |
|  | 5# | 15 |
|  | 6# | 15 |
|  | 7# | 14 |
|  | 8# | 19 |
|  | 9# | 15 |
|  | 10# | 20 |
|  | 11# | 18 |
|  | 12# | 19 |
|  | 13# | 14 |
|  | 14# | 16 |
|  | 15# | 14 |
|  | 16# | 14 |

Con: PBS-treated. CTX: CTX-treated.

Supplementary table 4. The original average score data of Hanging Wire test.

| **Hanging Wire test** | | |
| --- | --- | --- |
| Group | Time(sec) | Average score |
| Con (n=12) | 1-22s | 10 |
|  | 23-59s | 10.08 |
|  | 60-67s | 10 |
|  | 68-69s | 9.92 |
|  | 70-83s | 9.83 |
|  | 84-116s | 9.92 |
|  | 117-159s | 10 |
|  | 160-180s | 9.92 |
|  | | |
| CTX (n=16) | 1-20s | 10 |
|  | 21-34s | 9.94 |
|  | 35-115s | 10 |
|  | 116-153s | 9.94 |
|  | 154-180s | 10 |

Con: PBS-treated. CTX: CTX-treated.
